# Chemotherapy-free treatment for acute promyelocytic leukemia: the pediatric view of a revolutionary tale

**DOI:** 10.3389/fonc.2023.1135350

**Published:** 2023-04-14

**Authors:** Riccardo Masetti, Edoardo Muratore, Davide Leardini, Francesco Baccelli, Andrea Pession, Arcangelo Prete, Franco Locatelli

**Affiliations:** ^1^ Pediatric Oncology and Hematology “Lalla Seràgnoli”, IRCCS Azienda Ospedaliero-Universitaria di Bologna, Bologna, Italy; ^2^ Department of Medical and Surgical Sciences (DIMEC), University of Bologna, Bologna, Italy; ^3^ Pediatric Unit, IRCCS Azienda Ospedaliero-Universitaria di Bologna, Bologna, Italy; ^4^ Department of Pediatric Hematology/Oncology and Cell and Gene Therapy, IRCCS Ospedale Pediatric Bambino Gesù, Rome, Italy

**Keywords:** acute promyelocytic leukemia, pediatric, all-trans retinoic acid, arsenic trioxide, gemtuzumab-ozogamicin.

## Abstract

The addition of all-trans retinoic acid (ATRA) to the standard anthracycline-base chemotherapy has revolutionized the treatment of acute promyelocytic leukemia (APL) over the last decades, becoming a model for precision medicine. The protocols based on the combination of ATRA and chemotherapy allowed obtaining excellent response rates both for children and adults. However, the persistence of anthracycline chemotherapy as a backbone was a matter of concern for both acute and long-term complications. Efforts in reducing anthracycline cumulative dose or even eliminating anthracycline have been pursued in more recent pediatric protocols thanks to the introduction of arsenic trioxide (ATO). The impressive results of the ATRA/ATO combinations led to the introduction of protocols completely chemotherapy-free for standard-risk adult patients as the standard of care, whereas pediatric chemo-free protocols are still currently under evaluation. In this Review, we will critically retrace the history of this unique revolution in precision medicine, discussing the peculiar advantages for pediatric patients with APL.

## Introduction

Acute promyelocytic leukemia (APL) is a distinct subtype of acute myeloid leukemia (AML) accounting for 5-10% of all pediatric AML (1). Its incidence varies among geographical areas, with a higher prevalence in most Latino/Hispanic countries (2). APL is characterized by the typical balanced t(15;17) (q22;q21) translocation, recurrent in 95-98% of the cases (3). This translocation joins the promyelocytic leukemia (PML) gene located on chromosome 15 to the retinoic acid receptor alpha (RARα) gene on chromosome 17, resulting in an oncogenic fusion gene (PML-RARα) (4). In the remaining cases, other RARα rearrangements, cryptic insertions, or insertional viral mutagenesis have been described (5, 6). Pediatric APL is diagnosed mainly in late childhood, more than half of APL cases being diagnosed in children aged more than 10, while APL in infants is exceptionally rare (7). Females seem to be predominant in children compared to adults and the incidence of hyperleukocytosis, defined as white blood cells (WBC) >10 x 10^9^/L, is higher (8). Of note, despite these differences, pediatric APL has been treated with adult protocols for a long time. For many years, APL was considered as one of the most malignant forms of acute leukemia due to the very rapid fatal course secondary to associated severe coagulopathy in many patients. These early complications are associated with a high WBC count at diagnosis. For this reason, the risk assessment of APL is based on the number of blasts at diagnosis with patients with more or less than 10 x 10^9^/L WBC being considered as high and low risk, respectively (9).

In the last few years, APL treatment experienced a dramatic revolution. First, the introduction of all-trans retinoic acid (ATRA) in the chemotherapy protocols led to excellent response and survival rates in adults. This approach was subsequently applied to children with outstanding results (10). Second, arsenic trioxide acid (ATO) was introduced in the treatment of adult APL leading to the institution of completely chemotherapy-free protocols. In this view, a seminal randomized study by Lo-Coco and colleagues compared ATRA plus arsenic trioxide (ATO) vs all-trans retinoic acid (ATRA) plus anthracyclines as induction and consolidation treatment in the frontline setting for adult patients with low-risk APL. Although outcomes in both randomization arms were excellent, a statistically significant better event-free survival (EFS) and overall survival (OS) probability was observed in patients who received ATRA plus ATO alone (11). After these results, also pediatric hematologists started to adopt chemo-free treatments consisting only of ATRA and ATO with the addition of new target therapies, such as gemtuzumab-ozogamicin (GO) (12). In this review, we aim to critically review the development of this revolutionary approach with a particular focus on children.

## Drugs employed in chemotherapy-free regimens

### All-trans retinoic acid

**Figure 1:**
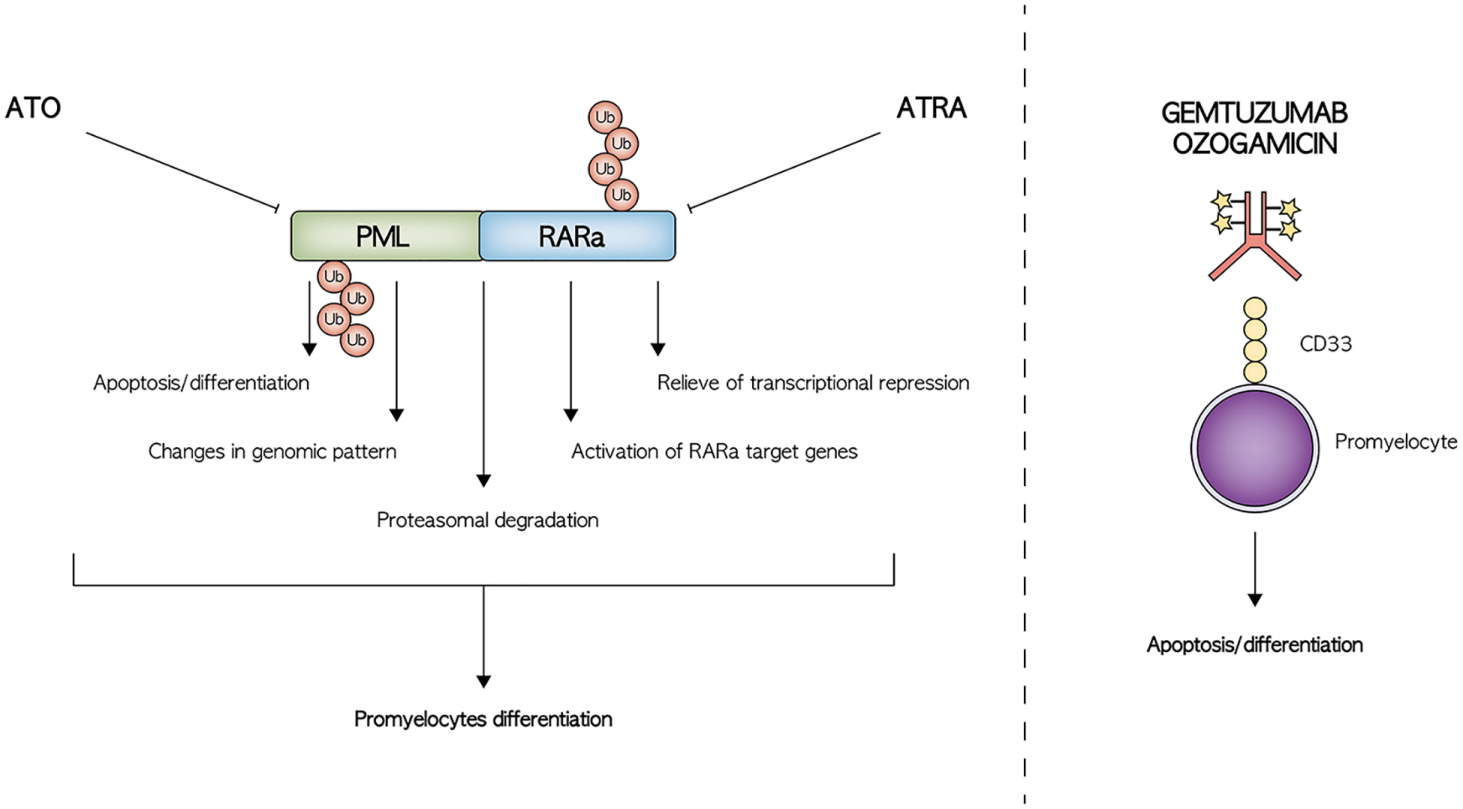
Schematic representation of the mechanism of action of the main drugs employed in the treatment of APL.

ATRA is an isomer of the 13-*cis* retinoic acid (RA) approved, at the time of discovery, for skin diseases such as psoriasis or acne (13). It was demonstrated in the early 80ies as an *in-vitro* differentiating agent for promyeloblasts (13). In the middle ‘80, an adult patient with APL treated with RA who obtained complete remission (CR) was described (14). ATRA was subsequently further tested, showing a higher differentiating capacity compared to the isomer 13-cis RA. ATRA was first used to treat a 5-year-old child with newly diagnosed APL in critical conditions, achieving CR and subsequently the treatment was extended to more patients and to the treatment of relapsed APL (15, 16). The mechanism of action of ATRA was later discovered, alongside the leukemogenic mechanism resulting from the PML-RARα translocation (17). In detail, the PML/RARα aberrant protein can form large homodimers repressing the expression of promyelocytes differentiating genes binding to the so-called retinoic acid response elements (12). Moreover, PML-RARα can also repress the RA downstream promoter *via* modifications in the DNA methylating enzymes (18). ATRA acts on both mechanisms, since on the one hand, induces PML/RARα aberrant protein degradation and on the other can relieve the transcriptional repression (19, 20) (**Figure 1**). A peculiar feature of ATRA treatment in children is the possible onset of a complication known as *pseudotumor cerebri* (21). ATRA can induce the production of cerebrospinal fluid and inhibits its reabsorption, resulting into increased endocranial pressure. This clinical manifestation can occur in 15% of pediatric patients, a proportion significantly higher than that of adults, presenting with headache, vision alterations, and cranial nerve dysfunction (22). In about 30% of cases, the discontinuation of ATRA is sufficient for the resolution of symptoms; for the remaining cases, medications including mannitol, acetazolamide, or topiramates may be used. Known risk factors for *pseudotumor cerebri* are the high doses of ATRA and obesity, defined as a body mass index higher than 28 kg/m^2^. Differentiation syndrome is another life-threatening condition that can occur following ATRA treatment in 5-20% of children. It is characterized by fever, hypotension, weight gain, pleural effusion, respiratory impairment, and renal failure, due to endothelial damage following promyelocyte differentiation. This complication can be responsible for early deaths if not promptly recognized and treated using corticosteroids. Of note, administration of ATRA after continuous exposure to the drug enhances its elimination, potentially impairing its efficacy, while intermittent schedule permits to achieve relatively high plasma drug exposure over the course of therapy (23). Intermittent schedule was also associated with better tolerability, especially in children, reducing the risk of ATRA-related neurotoxicity (24).

### Arsenic trioxide

ATO is a very old drug, already mentioned by Hippocrates for the treatment of skin ulcers, and used at the beginning of the 19^th^ century to treat several diseases. However, its use was abandoned for severe toxicities until the early 20^th^ century. In the 70ies, ATO was found to possess anticancer proprieties and was thus used to treat several tumors, including APL (25). Initial results were extremely encouraging with CR rate ranging from 65% in the first experiences to 90% in larger cohorts after the optimization of drug formulation (26–28). Recently, an oral formulation of ATO named Realgar-Indigo naturalis formula (RIF) was tested with similar clinical effects but easier use (29). ATO mechanism of action was later elucidated, although still not completely. Interestingly, ATO exerts two different effects based on its concentration. A high concentration of the drug induces apoptosis, while a low concentration induces differentiation of the promyelocytes (27). Both effects were shown to be restricted to cells carrying PML-RARα and wild-type PML, suggesting that ATO directly targets PML (30). Functional studies revealed that ATO can also modulate several genes regulated by ATRA, but its main function seems to be the induction of a deep change of proteome pattern in many intracellular pathways (19) (**Figure 1**). ATO toxicity profile includes differentiation syndrome and prolongation of the QT/QTc interval, which can occur in a variable percentage of cases between 10% and 17% (31, 32). This latter is potentially fatal, leading to *torsade de pointes*-type ventricular arrhythmia. Continuous cardiac monitoring should be performed on each patient and ATO discontinued when QTc interval increases over 450 msec. Of note, prolonged QTc has been described often without a clinical correlation and thus the indication of treatment discontinuation over 450 msec is a matter of debate (33). ATO can also results in hepatotoxicity with an increase in serum bilirubin, transaminases, or alkaline phosphatase and may require temporary suspension of the drug.

### Gemtuzumab-ozogamicin

GO is a recombinant humanized monoclonal antibody, directed against CD33, conjugated with a cytotoxic antibiotic called calicheamicin (34). Treatment with GO has been associated with an increased risk of hepatotoxicity and hepatic veno-occlusive disease, especially following HSCT, and other non-specific adverse events, such as myelosuppression, thrombocytopenia and tumor lysis syndrome (35, 36). CD33 is highly expressed on APL blasts and shows a highly homogeneous expression pattern, representing an optimal therapeutic target (37). Many clinical trials conducted both in children and adults have shown the efficacy of this antibody-drug conjugate in the setting of AML (38). Clinical data for GO in APL were first reported in early 2000. A patient with relapsed APL after upfront treatment with ATRA/ATO received GO, achieving CR (39). After this first case, many others were reported with encouraging results (40–42). *In vitro* studies later confirmed the anti-leukemic effect of GO against ATRA- and ATO-resistant APL cell lines (43).

### Adult data on chemotherapy-free treatment for APL

**Figure 2:**
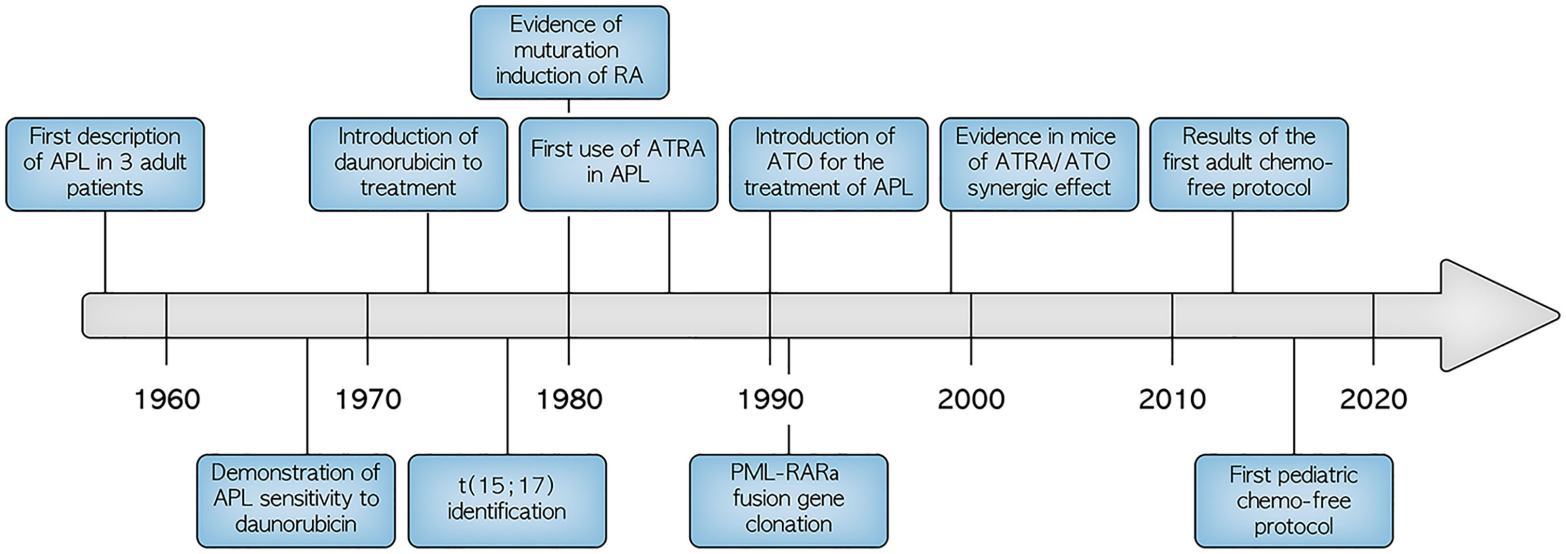
Timeline of the main steps in the development of chemo-free protocols for pediatric APL.

As already mentioned, specific therapeutic strategies for pediatric APL have been initially derived from adult trials. Chemotherapy has been historically used for the treatment of APL, and anthracyclines were recognized as the best suited class of drugs as early as 1970 (**Figure 2**). Chemotherapy with anthracyclines and cytarabine was considered the only treatment option for adult patients with APL until the late 1980s, when the introduction of all-*trans* retinoic acid (ATRA) led to an increase of remission rates up to 90-95%, together with a reduction of morbidity and mortality, mostly associated with severe coagulopathy (12, 44). Despite the dramatic improvements achieved in frontline therapy of APL with ATRA plus anthracycline-based regimens, relapses still occurred at a rate of approximately 10-20% (45). Moreover, these regimens were associated with significant toxicities due to severe myelosuppression, frequently associated with life-threatening infections and potentially serious late effects, including development of secondary MDS/AML and anthracycline-related myocardiopathy (46–50). The application of ATO since the early 1990s further improved the clinical outcome of refractory or relapsed, as well as newly of newly diagnosed APL^45^. In the randomized phase 3 APL0406 clinical trial conducted in 263 adults with newly diagnosed low- or intermediate-risk APL enrolled between 2007 and 2013, Lo Coco and colleagues compared induction and consolidation with ATRA and ATO vs ATRA and idarubicin plus maintenance with chemotherapy and ATRA. Low risk patients were defined as a white-cell count of no more than 10×10^9^/L and a platelet count of more than 40×10^9^/L at presentation, and intermediate risk level as a white-cell count lower than 10×10^9^/L and a platelet count of no more than 40×10^9^/L liter at presentation (11). The combination of ATRA and ATO resulted into statistically significant better EFS and OS rates at 50 months compared to the standard ATRA plus idarubicin therapy, 97.3% and 99.2% vs 80% and 92.6% respectively, with significantly reduced cumulative incidence of relapse and lower toxicity (11, 51). These data indicate that at least SR APL in adult patients can be cured with ATRA/ATO only and without chemotherapy. Notably, patients did not receive intrathecal therapy and, despite this, did not show increased incidence of CNS relapse. Recently, the AML17 randomized trial enrolled 235 patients including the ones with HR features in which GO was given at diagnosis to control the initial high WBC count if patients were randomized in the ATRA/ATO group. Compared to a standard ATRA and idarubicin regimen, ATRA/ATO resulted in higher EFS and reduced relapse rate at 4 years with no-difference in OS and quality of life (52). Long-term follow-up studies demonstrated the durability of the response and good short- and long-term tolerability without significant adverse effects (53–55).

### Results of ATRA-based regimens in pediatric APL

The combination of ATRA and chemotherapy in adults offered more efficacious results compared to pediatric studies with chemotherapy-only intensive regimens (56, 57). The use of ATRA in pediatric APL has thus been proposed as a potentially very effective approach since the beginning of 90s, with encouraging results in first small pediatric cohorts (9, 58, 59). Anthracycline-based regimens, often including high cumulative doses, were historically considered the mainstay of treatment for pediatric APL similar to adults, but their late effects have always represented a major and peculiar concern in children. Anthracyclines are known to cause a unique dose-dependent cumulative cardiotoxicity, with an increased risk of congestive heart failure in cancer survivors who received anthracyclines in their chemotherapeutic regimens. This dose-limiting late cardiotoxicity can cause severe morbidity and mortality, despite the progress in monitoring and preventing anthracycline cardiotoxicity. This is particularly important in in patients with long life expectancy, considering that the incidence of cardiac abnormalities increases with the time (23). These findings, associated with the promising results of the frontline administration of ATRA, led to a trend of anthracycline dose reduction in recent pediatric protocols in favor of ATRA administration along all the treatment. On the other hand, ATRA-related side effects, particularly *pseudotumor cerebri*, represented a peculiar concern in pediatric patients (60). The first large cohort of pediatric APL homogeneously diagnosed with genetic evidence of t(15;17) in blasts treated with ATRA-based regimen was derived from a multicenter Italian study by GIMEMA in association with AIEOP (AIDA-0493). The trial evaluated the use of ATRA combined with chemotherapy in newly diagnosed APL, enrolling also patients younger than 18 years. The treatment protocol consisted in ATRA and idarubicin induction therapy followed by three consolidation courses of anthracyclines. This resulted in the administration of high cumulative anthracycline dosage (650 mg/m^2^ of anthracycline daunorubicin equivalence). Molecular response was assessed by RT-PCR evaluating the PML-RARα transcript. Patients in complete molecular remission were randomized to either of four maintenance therapy arms, including 6-mercaptopurine and methotrexate, ATRA, chemotherapy with 6-mercaptopurine and methotrexate alternated to ATRA and no therapy. In children, ATRA was administered until the achievement of complete remission and for a maximum of 90 days, at a dose of 25 mg/m2/die in two doses, previously described as effective in adult population (61), with the aim of reducing the incidence of *pseudotumor cerebri*. Of the 107 eligible children who received induction with ATRA, 103 (96%) achieved a complete molecular remission. At the end of consolidation, ninety-four patients were evaluable for molecular analysis and 91 (97%) presented a complete molecular remission. The 10-year OS and EFS were 89% and 76%, respectively, and a WBC count greater than 10x10^9^/L at diagnosis resulted the strongest prognostic predictor on EFS (59% vs 83%) (62). Similar to the Italian experience, the national Spanish transplantation network (PETHEMA) reported outcomes of 66 children with molecularly-proven APL, who received induction therapy with ATRA and idarubicin and consolidation with three courses of anthracycline chemotherapy only, with a slightly reinforced doses of idarubicin in intermediate and high-risk patients. Maintenance therapy consisted of ATRA and oral chemotherapy. A high CR rate was reported (92%) with manageable toxicity and favorable long-term outcome (DFS 82% and OS 87% at 5 years). The high incidence of hyperleukocytosis in children with APL was confirmed to be associated with a higher risk of relapse (63). The North American Intergroup trial (INT0129) included children with previously untreated newly diagnosed APL, randomly assigned to receive daunorubicin plus cytarabine or ATRA as induction therapy. Similar to the previous studies, patients received ATRA until CR, or for a maximum of 90 days. Patients in CR after induction received two cycles of consolidation therapy with daunorubicin and cytarabine. A second randomization after consolidation assigned patients to receive a maintenance therapy with ATRA or to observation. The study compared rates of CR, DFS, OS and toxicity in patients who received ATRA for induction and/or maintenance compared to conventional chemotherapy. Fifty-three patients were evaluated, and a significant difference was shown in term of 5-year DFS from time of CR for patients who were randomized to ATRA, for induction or maintenance or both, compared to patients who never received ATRA (48% vs 0%) (64). These favorable results were confirmed in a European multicenter study including two consecutive trials (APL 93 and APL 2000) comparing disease characteristics and outcomes specifically in different age ranges, particularly children (age < 4 and 4-12 years), adolescents (13 to 18 years), and adults (> 18 years). All enrolled patients received ATRA in the induction phase, as monotherapy or in combination with chemotherapy. Interestingly, adolescents and children age > 4 years treated with ATRA and chemotherapy had outcomes comparable to those of adults, while younger children (12 patients) seemed to experience higher relapse rate (52% relapse compared to 18% in patients of 5-18 years), suggesting a less effective action of ATRA-based regimens in this particular category of patients (65). Subsequent protocols tried to adopt an anthracycline-sparing approach, as in the three consecutive AML-BFM 93/98/2004 trials, in which reduced cumulative anthracycline dose (350 mg/m^2^) was employed, combined with cytarabine and ATRA, with clinical outcomes achieved comparable to those reported in the AIDA 0493 protocol (66). A second multicenter Italian study (AIDA-2000) enrolled pediatric patients receiving the same induction therapy (ATRA 25 mg/m^2^/day and idarubicin) followed by a risk-adapted consolidation (67). Low- and intermediate-risk children, defined as in Lo Coco and colleagues, received three less intensive anthracycline-based courses plus ATRA, while for high-risk patients, consolidation was the same of AIDA-0493. Maintenance therapy consisted of standard daily 6-mercaptopurine and weekly methotrexate given for two years, plus ATRA administered for fifteen days every three months. The efficacy of ATRA plus idarubicin as induction was confirmed, and, for low/intermediate risk children, the anthracycline-based plus ATRA consolidation was equally effective as the previous cytarabine-containing regimen of AIDA-0493 (7, 68).

A more recent international study (ICC-APL-01) enrolled 258 European and South American children with newly diagnosed APL receiving induction treatment with ATRA and 3 doses of idarubicin. The primary aims of the trial were to reduce the cumulative anthracycline doses, while maintaining an excellent response and to investigate the efficacy of a risk-adapted consolidation therapy based on the initial WBC count. The therapeutical protocol included ATRA in induction, consolidation, and maintenance. For both standard- and high-risk patients, induction consisted of oral ATRA with pediatric dose of 25 mg/m2 per day, administered for 30 consecutive days and 3 doses of idarubicin. After induction, standard and high-risk patients received 2 or 3 consolidation blocks, respectively. Maintenance therapy included ATRA cycles, given for the first 14 days every 12 weeks with low-dose chemotherapy (oral 6-mercaptopurine daily and methotrexate weekly) and was given to all patients for 2 years. The cumulative dose of daunorubicin-equivalent anthracyclines administered in standard and high-risk patients was 355 mg/m^2^ and 405 mg/m^2^, respectively. The other main objective of the study was to evaluate systematically at different time points the PML-RARα transcript by PCR in order to investigate the value of MRD for predicting relapse and to guide the therapy. In fact, this approach permitted to treat with a third consolidation block, equal to that of the HR group, those SR patients who were positive for the PML-RARα transcript at the end of the second consolidation block. The transcript was re-evaluated at the end of consolidation therapy before the start of maintenance therapy. Resistant disease was defined as positive PCR after third consolidation block and was treated with salvage therapy consisting of ATO, GO and ATRA and, if refractory, with allogeneic hematopoietic stem cell transplant (HSCT). Results showed that complete molecular remission was obtained in 97% of patients, while 5-year OS and EFS were 94.6% and 79.9%, respectively in the whole cohort, 98.4% and 89.4% in standard risk patients and 84.3% and 74.2% in high-risk patients. These results were comparable to previous studies without any stratification of treatment on the basis of MRD and including higher dose of chemotherapy, confirming the effectiveness of ATRA combined with a risk-adapted consolidation strategy (69).

The management of CNS prophylaxis in pediatric ATRA based protocols varies among different studies. The incidence of CNS relapse was not homogeneously reported, and therefore any comparison regarding the efficacy of ATRA-based treatment and CNS prophylaxis could not be performed. In the protocols in which it is reported, CNS relapse is under 1% (63, 66). Heterogeneous approaches to CNS disease prophylaxis have been proposed in the different protocols described, with a wide range of strategies from no prophylaxis to several intrathecal therapies and cranial irradiation. Details of adopted prophylaxis regimens are reported in **Table 1**.

**Table 1:** Summary of pediatric studies using ATRA/ATO combination.

Reference	Enrollment period	Treatment for SR and HR	ATRA dose	ATO dose	Cumulative Anthracycline dose	Intrathecal CNS prophylaxis	Patients, n	EDR	CR end induction	CR end consolidation	OS	EFS	Median follow-up
Testi 2005, AIDA 0493, GIMEMA-AIEOP	1993-2000	Induction: ATRA + IDA. Consolidation: 3 chemotherapy courses. Maintenance (*random*): chemo vs ATRA vs chemo-ATRA vs no therapy	25 mg/m^2^/day	/	650 mg/m^2^	/	107	3.7%	96% (hematological)	97% (molecular)	89%	76%	79
Ortega 2005, PETHEMA	1996-2004	Induction: ATRA + IDA. Consolidation: 3 chemotherapy courses. Maintenance: chemo + ATRA (increased idarubicin dose in HR patients)	25 mg/m^2^/day	/	650 mg/m2 (+20 mg/m2 idarubicin in HR)	/	66	7.6%	92% (hematological)	97% (molecular)	87%	[DFS] 82%	39
Gregory 2009, INT0129, CCG-POG	1992-1995	Induction (*random*): chemo (DNR + ARA-C) vs ATRA. Consolidation 2 courses. Maintenance (*random*): ATRA vs no therapy	ATRA 45 mg/m^2^/day	/	225-495 mg/m2	/	53 (27 ATRA / 26 chemo)	3.7% (ATRA) vs 11.5 % (chemo)	81% (ATRA) vs 65% (chemo) (hematological)	/	69.6% ATRA: 73% Chemo: 65%	[DFS] 41% ATRA: 49% Chemo: 29%	60
Bally 2012, European APL group, APL 93 and 2000 trials	1993-2004	*APL 93 trial.* Induction: risk stratification according to WBC count (< / > 5x10^9^/L); in low risk (*random*): ATRA followed by chemotherapy (DNR + ARA-C) vs early addition of chemotherapy. Consolidation: 2 chemotherapy courses. Maintenance (*random*) ATRA vs chemo vs ATRA + chemo vs no therapy [for 2 years] *APL 2000 trial.* Induction: risk stratification = APL 93; in low risk [*random*): with or without ARA-C. ARA-C dose doubled in high risk	ATRA 45 mg/m^2^/day	/	495 mg/m2	*APL 2000 trial:* MTX and ARA-C x1 during induction and x4 during consolidation (if WBC > 10x 10^9^/L)	84 (26 ≤12 y/o / 58 13-18 y/o)	/	92% (≤12 y/o) 100% (13-18 years) (morphological)	/	80,4% (≤12 y/o) 93.6% (13-18 years)	/	60
Creutzig 2010, AML-BFM, AML-BFM 93-98-2004 protocol	1993-2007	ATRA during induction, consolidation and maintenance. Patients received uniform AML-BFM SR therapy (not stratified by WBC count): induction [ARA-C, DNR and VP-16 - ADE), consolidation (2 cycles of 6-week therapy with seven different drugs or two blocks AI (ARA-C + idarubicin) + hAM (HD-ARA-C + mitoxantrone); intensification (HD ARA-C + VP-16 + cranial irradiation); maintenance: oral chemo	25 mg/m^2^/day	/	300 mg/m2 (trial 93), 320–350 mg/m2 (trial 98), and 350–410 mg/m2 (trial 2004).	ARA-C x11 + cranial irradiation after intensification	81	7.4%	93%	/	89 (5 y) and 82 (10 y)	73 (5 y) and 65 (10 y)	120
Lo-Coco 2010, Testi 2010, GIMEMA-AIEOP AIDA 2000	2000-2009	Induction = AIDA 0493 Consolidation: risk-adapted according to Sainz criteria: low and intermediate risk: less intensive anthracycline-based courses plus ATRA high risk patients = AIDA 0493 (3 chemotherapy courses) + ATRA Maintenance: daily 6-MP and weekly MTX for 2 years + ATRA for 15 days every 3 months	25 mg/m^2^/day	/	650 mg/m2	/	123	0%	100% (hematological)	/	96% LR/IR: 95.6% HR: 96.8%	82.5% LR/IR: 82.7% HR: 82.3%	72
Testi 2018, International Consortium for Childhood APL , ICC-APL-01	2008-2017	Patients stratified into SR/HR according to the baseline WBC count </> 10x10^9^/L Induction: SR and HR ATRA + idarubicin. Consolidation: SR (2 courses) and HR (3 courses) + ATRA [Course 1: ARA-C and MTZ; Course 2: idarubicin; Course 3: HD-ARA-C and idarubicin]. Maintenance: oral 6-MP daily and MTX weekly + ATRA for 14 days every 12 weeks); 2 years.	25 mg/m^2^/day	/	SR: 355 mg/m2 HR: 405 mg/m2	ARA-C at the start of each consolidation block	258	3.1%	97% (hematological)	94% (molecular)	94.6% SR: 98.4 HR: 89.4 0-5y: 100 6-12y: 94.3 13-18y: 93	79.9% SR: 84.3 HR: 74.2 0-5y: 78 6-12y: 80.9 13-18y: 79.4	52
Cheng 2013, The Affiliated People’s Hospital of Peking University, Beijing, China	1998-2011	Induction ATRA/ATO Consolidation with at least two cycles comprising daunorubicin, Ara-c, Vp16, homoharringtonine and ATRA/ATO. Maintenance with ATRA and mercaptopurine	20–40 mg/m^2^/day	0.16 mg/kg/day	<350 mg/m^2^	MTX, dexamethasone and ARA-C (if WBC > 10x10^9^/L or CNS involvement)	43	4,7%	95,3% (hematological)	95,3% (hematological)	95,2%	92,5%	75
Kutny 2017, COG AAML0631Trial	2009-2012	Induction: ATRA only Consolidation ATRA/ATO and 2 chemotherapy cycles in SR or 3 cycles in HR Maintenance: ATRA plus chemotherapy.	25 mg/m^2^/day	0.15 mg/kg/day	SR: 335 mg/m^2^ HR: 385 mg/m^2^	ARA-C SR: x3 HR: x4	SR: 66 HR: 35	0%	81% (hematological)	100% (molecular)	SR: 98% HR: 86%	SR: 95% HR: 83%	44,8
Creutzig 2017, BFM	2013-2016	Modified APL0406 schedule: A later start of ATO on day 10, lower ATRA dose and a 1- week break of ATRA after the first 14 days.	25 mg/m^2^/day	0.15 mg/kg/day	0	ARA-C every 4 weeks starting at day 10 or after blast cell reduction	11	0%	100% (molecular)	100% (molecular)	/	/	29
Strocchio 2018, AIEOP	2014-2017	Modified APL0406 schedule: lower ATRA dose	25 mg/m^2^/day	0.15 mg/kg/day	0	/	18	0%	100% (hematological)	100% (molecular)	/	/	24
Garcia Spezza 2019, French SFCE	2015-2018	SR: induction ATRA/ATO HR patients: idarubicin added at days 1 and 3.	25 mg/m^2^/day	0.15 mg/kg/day	120 mg/m^2^	/	21	0%	100% (molecular)	100% (molecular)	100%	100%	17
Li 2021, Yinzhou Hospita,, Ningbo Medical Center, Lihuili Hospital ,Hwa Mei Hospital	2008-2018	Induction and consolidation with ATRA/ATO only	25 mg/m^2^/day	0.16 mg/kg/day	0	MTX, dexamethasone and ARA-C CR (SR: x4; HR x6)	197	5,9%	94,1% (molecular)	94,1% (molecular)	94,1%	100%	49
Hiroyuki 2022, preliminary results of the JPLSG AML-P13 Study	2014-2018	SR: induction consisting of ATRA, cytarabine, and daunorubicin, consolidation with ATRA/ATO, maintenance with intermittent ATRA HR (failure to achieve hematological remission after induction): induction consisting of ATRA, cytarabine, and daunorubicin, consolidation with intensified schedule of ATO, followed by gemtuzumab ozogamicin, maintenance with 6-mercaptopurine and methotrexate	/	/	/	/	25	/	92% (hematological)	100% (molecular)	100%	96,3 %	36
Zheng 2022, CCLG-APL2016 Protocol	2016-2018	SR: induction, consolidation and maintenance with ATRA/ATO only HR: low dose anthracycline added in induction and consolidation	25 mg/m^2^/day	0.16 mg/kg/day	SR: 0 HR: 200-300 mg/ m^2^	/	SR: 107 HR: 86	SR: 1% HR: 5%	/	SR: 99% (molecular) HR: 95% (molecular)	SR: 99% HR: 95%	SR: 97% HR: 90%	28,9
Kutny 2022, COG AAML1331 Trial	2015-2019	SR: induction and consolidation with ATRA/ATO only, no maintenance HR: idarubicin added during induction, no maintenance	25 mg/m^2^/day	0.16 mg/kg/day	SR: 0 HR: 240 mg/ m^2^	MTX, hydrocortisone and ARA-C twice weekly until CSF negative plus one week.. Additional doses (x6) during consolidation. Only in patients with CNS leukemia or hemorrhage	SR: 98 HR: 56	SR: 1,0% HR: 0%	100% (molecular)	100% (molecular)	SR: 99% HR: 100%	SR: 98% HR: 96,6%	24,7

"/" means that the data was not reported.

### Preliminary data on the use of the combination of ATRA and ATO in pediatric APL

Studies in adult APL showed that with the combination of ATRA and ATO leads to excellent results with lower toxicities compared to the therapy based on chemotherapy with ATRA (11). In the pediatric setting, the use of ATO was initially introduced for the treatment of patients with refractory/relapsed PML-RARα positive APL, with promising results (70, 71). The combination ATRA/ATO was then translated to the first-line therapy. A Chinese single-center study reported data about the use of ATO combined with ATRA as induction and consolidation therapy in combination with chemotherapy in newly diagnosed childhood APL Forty-three children, eleven of which classified in the HR group, treated with ATRA/ATO were compared with 25 pediatric patients (2 HR) previously treated with ATRA alone as induction therapy. The ATRA/ATO group achieved complete remission (CR) rates of 95.4% compared to 80% in ATRA-alone cohort. With a median follow-up time of 75 months, EFS and OS were 92.5% and 95.3% respectively in the ATRA/ATO group, significantly higher compared with the ATRA group. Of note, one patient had CNS relapse during consolidation chemotherapy (72). The COG AAML0631 trial enrolled 101 patients (66 SR and 35 HR). Both groups received ATRA only induction therapy, consolidation with ATRA/ATO and chemotherapy and maintenance with ATRA plus chemotherapy. The only difference in the two groups was the number of chemotherapy consolidation cycles, 2 versus 3. As a result, the cumulative anthracycline dose was 335 mg/m^2^ of cumulative daunorubicin equivalents for SR patients and 385 mg/m^2^ in the HR group. In the HR group 0.8% of patients died during induction, due to coagulopathy and differentiation syndrome, while no patients experienced an early death event in the SR group. Of the three relapses recorded, one in the SR group was a combined CNS and bone marrow, while in the HR group one was combined and one an isolated bone marrow relapse. The 3-year OS and EFS rate of 98% and 95% respectively were achieved for SR patients, while in the HR group the 3-year OS and EFS were 86% and 83%, respectively (73). Therefore, EFS for SR patients was noninferior to that of patients in the AIDA 0493 historical control, which used a significantly higher anthracycline dose and did not include ATO consolidation (73). A first international series of 11 pediatric patients treated with ATRA/ATO only in induction and consolidation phases was described by the AML-BFM group. All patients were treated according to a protocol similar to the adult APL0406, with lower ATRA dose, delayed start of ATO and an intermittent schedule. In all cases a CR was obtained after a median time of 10 weeks, without early death events (31). A chemotherapy-free approach was applied in 18 Italian children with newly diagnosed APL, of whom 89% were SR, using a modified APL0406 schedule with lower ATRA dose. All patients achieved molecular CR and were alive at a median follow-up of 24 months, with good tolerance to the therapy (32). These results were confirmed in a French cohort of 21 children, with all patients achieving molecular remission with a median of 8 weeks. OS and EFS of 100% were achieved after a median follow up of 17 months. Notably, the French protocol include two doses of idarubicin in HR patients (74). Comparable results were reported in another Chinese cohort of 17 pediatric patients with newly diagnosed APL treated with ATRA/ATO only, showing an OS and EFS of 94.1% and 100%, respectively. One patient in this protocol died during induction due to intracranial hemorrhage (75). Preliminary results on 27 children were reported from the Japanese protocol JPLSG AML-P13. Patients received 2 courses of induction consisting of ATRA, cytarabine, and daunorubicin. Patients with hematological CR were considered SR and received 3 intensification cycles of ATRA/ATO, followed by ATRA maintenance therapy, while patients non in CR were considered HR and received intensified schedule of ATO, followed by GO and a maintenance of 6-mercaptopurine and methotrexate. Molecular remission rate was 100% in both groups after intensification and no patient experienced relapse. The 3-year EFS rate and 3-year OS rate in full analysis set were 96.3% and 100.0%, respectively (76). Interestingly, the ATRA/ATO combined approach was also applied as a stand-alone therapy in children with relapsed APL with promising results in several case reports (77).

### Clinical trial assessing the combination of ATRA and ATO for first line treatment of pediatric APL

Given the revolutionary results of the adult trial regarding the treatment of SR APL with chemo-free regimens and the encouraging preliminary data on the use of ATRA/ATO in children, two clinical trials have been performed in pediatric patients assessing the combination of ATRA and ATO alone for the treatment of newly diagnosed APL. The CCLG-APL2016 multicenter trial enrolled 193 children from 38 Chinese centers. In SR patients, induction, consolidation and maintenance therapy were performed using ATRA/ATO only, while for HR patients low dose anthracycline (200-300 mg/m^2^ of cumulative daunorubicin equivalents) was added in the induction and consolidation phase. Interestingly, ATO was administered either intravenously or with an oral formulation depending on the investigator’s choice. In 107 patients allocated to the SR group, the 2-year OS and EFS were 99% and 97% respectively, whereas in the high-risk group the corresponding value were was 95% and 90%. Only one patient in the HR group had combined CNS and bone marrow relapse despite the absence of intrathecal prophylaxis in the protocol. ATRA/ATO regimen was well tolerated without treatment-related death, and no retention of ATO in plasma, urine, hair, and nail was detected 6 months after the cessation of treatment (78). 154 pediatric patients from 85 pediatric oncology centers were enrolled in the COG AAML1331 trial and compared to an historical control group from the AAML0631 study. Patients in the SR received ATRA/ATO only as induction and consolidation therapy, while HR patients received 4 doses of idarubicin during induction and 14 days of dexamethasone therapy to prevent differentiation syndrome. No maintenance was administered in any patient. Therapy was well tolerated in both risk groups; 98 children were included in the SR group, with 98% 2-year EFS rate and 99% 2-year OS rate. The HR group had a 2-year EFS and OS rate of 96.6% and 100%, respectively. Despite using intrathecal therapy only in patients with CNS leukemia or hemorrhage (see **Table 1**), only one patient in the SR group experienced a combined bone marrow and CNS leukemia, confirming that pediatric APL patients who received arsenic trioxide may not require intrathecal treatment (68). These outcomes were noninferior compared to the historical comparator group treated with a combination of ATRA/ATO and chemotherapy (73). The advantages of the AAML1331 regimen compared to AAML0631 included shorter treatment duration, lower exposure to anthracycline and intrathecal chemotherapy, and fewer days of hospitalization (68). Two trials are currently ongoing assessing the application of totally chemo-free treatment protocols for newly diagnosed pediatric APL. The ICC APL 02 international trial (NCT04793919) applies an induction phase with ATRA/ATO alone for both SR and HR patients and a consolidation composed of four 28-day long ATO and seven 14-day long ATRA courses. For HR patients only, two doses of GO at 3 mg/m^2^ are administered on days 2 and 4 of induction therapy. A chemo-free treatment protocol is under investigation as a Phase II study at MD Anderson (NCT01409161) as well. Patients, regardless of the risk group, receive as induction ATRA/ATO, and only high-risk patients receive GO once at weeks 1 and 4 at 9 mg/m^2^. Patients achieving CR receive as consolidation ATO during weeks 1-4, 9-12, 17-20, and 25-28 and ATRA for 2 weeks on and 2 weeks off for a total of 4 courses. This intermittent schedule ensures the efficacy of ATRA limiting the increasing drug elimination typical of continuous administration and lowering the risk of neurotoxicity.

## Conclusions

Treatment for APL is nowadays considered a prototypal model of precision medicine. As for other subtypes of pediatric acute leukemias (79), APL, in the past a highly fatal disease especially in the first few days after diagnosis, slowly became highly curable. This success is due to the development and application to the patient bedside of translational research and well-designed clinical trials. Translation of adult data to the pediatric population has been successful in pediatric APL; however, some differences persist with particular consideration to the long-term sequelae. Pediatric patients strongly benefit from chemo-free protocols considering their longer life-expectancy. Despite the impressive 2-year outcome reported in the CCLG-APL2016 and the COG AAML1331 trials for chemo-free treatment of SR patients, longer follow-up data are needed. Definitive data are also needed for the use of GO in the treatment of APL, with a particular focus on systemic short- and long-term toxicities. Moreover, the implementation of new drugs, such as the oral formulation of ATO, will further improve the quality of life of patients with APL during treatment. The ongoing pediatric ATRA/ATO trials, the ICC APL 02 and the MD Anderson study employing only ATRA/ATO protocols with the addition of GO in the HR group will provide important information regarding the effectiveness of chemo-free protocols even for HR patients and the real incidence of long-term sequelae.

## Author contributions

RM designed the review. EM, DL and FB wrote the original draft. EM and FB designed the table. DL designed the figures APe, APr, FL and RM critically reviewed the manuscript. All authors contributed to the article and approved the submitted version.

## References

[1] Testi AM D’Angiò M Locatelli F Pession A Coco F. Lo. acute promyelocytic leukemia (APL): Comparison between children and adults. Mediterr J Hematol Infect Dis (2014) 6:2014032. doi: 10.4084/mjhid.2014.032 PMC4010611 24804005

[2] Douer D Preston-Martin S Chang E Nichols P Watkins K Levine A. High frequency of acute promyelocytic leukemia among latinos with acute myeloid leukemia. Blood (1996) 87:308–13. doi: 10.1182/blood.V87.1.308.308 8547657

[3] Rowley JD Golomb HM Dougherty C. 15/17 translocation, a consistent chromosomal change in acute promyelocytic leukaemia. Lancet (1977) 1:549–50. doi: 10.1016/S0140-6736(77)91415-5 65649

[4] Zwaan CM Kolb EA Reinhardt D Abrahamsson J Adachi S Aplenc R. Collaborative efforts driving progress in pediatric acute myeloid leukemia. J Clin Oncol (2015) 33:2949–62. doi: 10.1200/JCO.2015.62.8289 PMC4567700 26304895

[5] Astolfi A Masetti R Indio V Bertuccio SN Messelodi D Rampelli S. Torque teno mini virus as a cause of childhood acute promyelocytic leukemia lacking PML/RARA fusion. Blood (2021) 138:1773–7. doi: 10.1182/blood.2021011677 34432867

[6] Cicconi L Testi AM Montesinos P Rego E Zhu HH Takahashi H. Characteristics and outcome of acute myeloid leukemia with uncommon retinoic acid receptor-alpha (RARA) fusion variants. Blood Cancer J (2021) 11:1–4. doi: 10.1038/s41408-021-00561-w 34657125 PMC8520532

[7] Lo-Coco F Avvisati G Vignetti M Breccia M Gallo E Rambaldi A. Front-line treatment of acute promyelocytic leukemia with AIDA induction followed by risk-adapted consolidation for adults younger than 61 years: Results of the AIDA-2000 trial of the GIMEMA group. Blood (2010) 116:3171–9. doi: 10.1182/blood-2010-03-276196 20644121

[8] de Botton S lo Coco F Martín G Avvisati G Rayón C Barbui T. Outcome of childhood acute promyelocytic leukemia with all-trans-retinoic acid and chemotherapy. J Clin Oncol (2004) 22:1404–12. doi: 10.1200/JCO.2004.09.008 15084614

[9] Sanz MA Coiteux V Chevret S Rayon C Vilmer E Sanz M. Definition of relapse risk and role of nonanthracycline drugs for consolidation in patients with acute promyelocytic leukemia: a joint study of the PETHEMA and GIMEMA cooperative groups. Blood (2000) 96:1247–53. 10942364

[10] Gurnari C Voso MT Girardi K Mastronuzzi A Strocchio L. Acute promyelocytic leukemia in children: A model of precision medicine and chemotherapy-free therapy. Int J Mol Sci (2021) 22:1–13. doi: 10.3390/ijms22020642 PMC7826974 33440683

[11] Lo-Coco F Avvisati G Vignetti M Thiede C Orlando SM Iacobelli S. Retinoic acid and arsenic trioxide for acute promyelocytic leukemia. New Engl J Med (2013) 369:111–21. doi: 10.1056/NEJMoa1300874 23841729

[12] Wang ZY Chen Z. Acute promyelocytic leukemia: From highly fatal to highly curable. Blood (2008) 111:2505–15. doi: 10.1182/blood-2007-07-102798 18299451

[13] Runde V Aul C Südhoff T Heyll A Schneider W. Retinoic acid in the treatment of acute promyelocytic leukemia: inefficacy of the 13-cis isomer and induction of complete remission by the all-trans isomer complicated by thromboembolic events. Ann Hematol (1992) 64:270–2. doi: 10.1007/BF01695469 1637880

[14] Daenen S Vellenga E van Dobbenburgh OA Halie MR. Retinoic acid as antileukemic therapy in a patient with acute promyelocytic leukemia and aspergillus pneumonia. Blood (1986) 67:559–61. doi: 10.1182/blood.V67.2.559.559 3455829

[15] Huang ME Ye YC Chen SR Chai JR Lu JX Zhoa L. Use of all-trans retinoic acid in the treatment of acute promyelocytic leukemia. Blood (1988) 72:567–72. doi: 10.1182/blood.V72.2.567.567 3165295

[16] Huang ME Ye YC Chen SR Zhao JC Gu LJ Cai JR. All-trans retinoic acid with or without low dose cytosine arabinoside in acute promyelocytic leukemia. report of 6 cases. Chin Med J (Engl) (1987) 100:949–53. 3133168

[17] Masetti R Biagi C Zama D Vendemini F Martoni A Morello W. Retinoids in pediatric onco-hematology: The model of acute promyelocytic leukemia and neuroblastoma. Adv Ther (2012) 29:747–62. doi: 10.1007/s12325-012-0047-3 22941525

[18] Lo-Coco F Ammatuna E. The biology of acute promyelocytic leukemia and its impact on diagnosis and treatment. Hematol Am Soc Hematol Educ Program (2006) 514:156–61. doi: 10.1182/asheducation-2006.1.156 17124055

[19] Zheng P-Z Wang KK Zhang QY Huang QH Du YZ Zhang QH. Systems analysis of transcriptome and proteome in retinoic acid/arsenic trioxide-induced cell differentiation/apoptosis of promyelocytic leukemia. Proc Natl Acad Sci U.S.A. (2005) 102:7653–8. 10.1073/pnas.0502825102 PMC1140456 15894607

[20] Nervi C Ferrara FF Fanelli M Rippo MR Tomassini B Ferrucci PF. Caspases mediate retinoic acid-induced degradation of the acute promyelocytic leukemia PML/RARalpha fusion protein. Blood (1998) 92:2244–51. 9746761

[21] Masetti R Vendemini F Zama D Biagi C Gasperini P Pession A. All-trans retinoic acid in the treatment of pediatric acute promyelocytic leukemia. Expert Rev Anticancer Ther (2012) 12:1191–204. doi: 10.1586/era.12.101 23098119

[22] Coombs CC DeAngelis LM Feusner JH Rowe JM Tallman MS. Pseudotumor cerebri in acute promyelocytic leukemia patients on intergroup protocol 0129: Clinical description and recommendations for new diagnostic criteria. Clin Lymphoma Myeloma Leuk (2016) 16:146–51. doi: 10.1016/j.clml.2015.11.018 PMC5028896 26724834

[23] Lanvers C Reinhardt D Dübbers A Wagner-Bohn A Creutzig U Ritter J. Pharmacology of all-trans-retinoic acid in children with acute promyelocytic leukemia. Med Pediatr Oncol (2003) 40:293–301. doi: 10.1002/mpo.10257 12652617

[24] Adamson PC Bailey J Pluda J Poplack DG Bauza S Murphy RF. Pharmacokinetics of all-trans-retinoic acid administered on an intermittent schedule. J Clin Oncol (1995) 13:1238–41. doi: 10.1200/JCO.1995.13.5.1238 7738627

[25] Zhu J Chen Z Lallemand-Breitenbach V de Thé H. How acute promyelocytic leukaemia revived arsenic. Nat Rev Cancer (2002) 2:705–13. doi: 10.1038/nrc887 12209159

[26] Chen GQ Shi XG Tang W Xiong SM Zhu J Cai X. Use of arsenic trioxide (As2O3) in the treatment of acute promyelocytic leukemia (APL): I. As2O3 exerts dose-dependent dual effects on APL cells. Blood (1997) 89:3345–53. 9129041

[27] Shen ZX Chen GQ Ni JH Li XS Xiong SM Qiu QY. Use of arsenic trioxide (As2O3) in the treatment of acute promyelocytic leukemia (APL): II. clinical efficacy and pharmacokinetics in relapsed patients. Blood (1997) 89:3354–60. doi: 10.1182/blood.V89.9.3354 9129042

[28] Niu C Yan H Yu T Sun HP Liu JX Li XS. Studies on treatment of acute promyelocytic leukemia with arsenic trioxide: remission induction, follow-up, and molecular monitoring in 11 newly diagnosed and 47 relapsed acute promyelocytic leukemia patients. Blood (1999) 94:3315–24. doi: 10.1182/blood.V94.10.3315.422k16_3315_3324 10552940

[29] Yang M-H Wan WQ Luo JS Zheng MC Huang K Yang LH. Multicenter randomized trial of arsenic trioxide and realgar-indigo naturalis formula in pediatric patients with acute promyelocytic leukemia: Interim results of the SCCLG-APL clinical study. Am J Hematol (2018) 93:1467–73. doi: 10.1002/ajh.25271 PMC6282847 30160789

[30] Chen GQ Zhu J Shi XG Ni JH Zhong HJ Si GY. *in vitro* studies on cellular and molecular mechanisms of arsenic trioxide (As2O3) in the treatment of acute promyelocytic leukemia: As2O3 induces NB4 cell apoptosis with downregulation of bcl-2 expression and modulation of PML-RAR alpha/PML proteins. Blood (1996) 88:1052–61. doi: 10.1182/blood.V88.3.1052.1052 8704214

[31] Strocchio L Gurnari C Santoro N Putti MC Micalizzi C Zecca M. Arsenic trioxide and all-trans retinoic acid treatment for childhood acute promyelocytic leukaemia. Br J Haematol (2019) 185:360–3. doi: 10.1111/bjh.15507 30028005

[32] Garcia Spezza E Brethon B Petit A Mazingue F Gandemer V Boissel N. Tolerance to arsenic trioxide combined with all-trans-retinoic acid in children with acute promyelocytic leukaemia in France. Br J Haematol (2020) 188:170–3. doi: 10.1111/bjh.16364 31808943

[33] Roboz GJ Ritchie EK Carlin RF Samuel M Gale L Provenzano-Gober JL. Prevalence, management, and clinical consequences of QT interval prolongation during treatment with arsenic trioxide. J Clin Oncol (2014) 32:3723–8. doi: 10.1200/JCO.2013.51.2913 25245447

[34] Stasi R. Gemtuzumab ozogamicin: an anti-CD33 immunoconjugate for the treatment of acute myeloid leukaemia. Expert Opin Biol Ther (2008) 8:527–40. doi: 10.1517/14712598.8.4.527 18352855

[35] Leopold LH Berger MS Feingold J. Acute and long-term toxicities associated with gemtuzumab ozogamicin (Mylotarg) therapy of acute myeloid leukemia. Clin Lymphoma (2002) 2(Suppl 1):S29–34. doi: 10.3816/clm.2002.s.006 11970768

[36] Guglielmi C Martelli MP Diverio D Fenu S Vegna ML Cantù-Rajnoldi A. Immunophenotype of adult and childhood acute promyelocytic leukaemia: correlation with morphology, type of PML gene breakpoint and clinical outcome. a cooperative Italian study on 196 cases. Br J Haematol (1998) 102:1035–41. doi: 10.1046/j.1365-2141.1998.00871.x 9734655

[37] Gamis AS Alonzo TA Meshinchi S Sung L Gerbing RB Raimondi SC. Gemtuzumab ozogamicin in children and adolescents with *de novo* acute myeloid leukemia improves event-free survival by reducing relapse risk: Results from the randomized phase iII children’s oncology group trial AAML0531. J Clin Oncol (2014) 32:3021–32. doi: 10.1200/JCO.2014.55.3628 PMC4162498 25092781

[38] Petti MC Pinazzi MB Diverio D Romano A Petrucci MT De Santis S. Prolonged molecular remission in advanced acute promyelocytic leukaemia after treatment with gemtuzumab ozogamicin (Mylotarg CMA-676). Br J Haematol (2001) 115:63–5. doi: 10.1046/j.1365-2141.2001.03091.x 11722411

[39] Tsimberidou A-M Estey E Whitman GJ Dryden MJ Ratnam S Pierce S. Extramedullary relapse in a patient with acute promyelocytic leukemia: successful treatment with arsenic trioxide, all-trans retinoic acid and gemtuzumab ozogamicin therapies. Leuk Res (2004) 28:991–4. doi: 10.1016/j.leukres.2004.01.004 15234578

[40] Schwarz J Marková J Peková S Trnková Z Sponerová D Cetkovský P. A single administration of gemtuzumab ozogamicin for molecular relapse of acute promyelocytic leukemia. Hematol J (2004) 5:279–80. doi: 10.1038/sj.thj.6200367 15167917

[41] Lo-Coco F Cimino G Breccia M Noguera NI Diverio D Finolezzi E. Gemtuzumab ozogamicin (Mylotarg) as a single agent for molecularly relapsed acute promyelocytic leukemia. Blood (2004) 104:1995–9. doi: 10.1182/blood-2004-04-1550 15187030

[42] Takeshita A Shinjo K Naito K Matsui H Sahara N Shigeno K. Efficacy of gemtuzumab ozogamicin on ATRA- and arsenic-resistant acute promyelocytic leukemia (APL) cells. Leukemia (2005) 19:1306–11. doi: 10.1038/sj.leu.2403807 15920495

[43] Fenaux P Chastang C Chevret S Sanz M Dombret H Archimbaud E. A randomized comparison of all transretinoic acid (ATRA) followed by chemotherapy and ATRA plus chemotherapy and the role of maintenance therapy in newly diagnosed acute promyelocytic leukemia. Blood (1999) 94:1192–200. doi: 10.1182/blood.V94.4.1192 10438706

[44] Yanada M. Treatment for relapsed acute promyelocytic leukemia. Ann Hematol (2022) 1:1–8. doi: 10.1007/s00277-022-04954-0 35972562

[45] Adès L Guerci A Raffoux E Sanz M Chevallier P Lapusan S. Very long-term outcome of acute promyelocytic leukemia after treatment with all-trans retinoic acid and chemotherapy: the European APL group experience. Blood (2010) 115:1690–6. doi: 10.1182/blood-2009-07-233387 20018913

[46] Burnett AK Hills RK Grimwade D Jovanovic Jv Craig J McMullin MF. Inclusion of chemotherapy in addition to anthracycline in the treatment of acute promyelocytic leukaemia does not improve outcomes: results of the MRC AML15 trial. Leukemia (2013) 27:843–51. doi: 10.1038/leu.2012.360 23222369

[47] Sanz MA. Management of acute promyelocytic leukemia: recommendations from an expert panel on behalf of the European LeukemiaNet. Blood (2009) 113:1875–91. doi: 10.1182/blood-2008-04-150250 18812465

[48] Montesinos P González JD González J Rayón C de Lisa E Amigo ML. Therapy-related myeloid neoplasms in patients with acute promyelocytic leukemia treated with all-trans-retinoic acid and anthracycline-based chemotherapy. J Clin Oncol (2010) 28:3872–9. doi: 10.1200/JCO.2010.29.2268 20625122

[49] Sanz MA Grimwade D Tallman MS Lowenberg B Fenaux P Estey EH. Management of acute promyelocytic leukemia: updated recommendations from an expert panel of the European LeukemiaNet. Blood (2019) 133:1630–43. doi: 10.1182/blood-2019-01-894980 PMC6509567 30803991

[50] Platzbecker U Avvisati G Cicconi L Thiede C Paoloni F Vignetti M. Improved outcomes with retinoic acid and arsenic trioxide compared with retinoic acid and chemotherapy in non-high-risk acute promyelocytic leukemia: Final results of the randomized Italian-German APL0406 trial. J Clin Oncol (2017) 35:605–12. doi: 10.1200/JCO.2016.67.1982 27400939

[51] Burnett AK Russell NH Hills RK Bowen D Kell J Knapper S. Arsenic trioxide and all-trans retinoic acid treatment for acute promyelocytic leukaemia in all risk groups (AML17): results of a randomised, controlled, phase 3 trial. Lancet Oncol (2015) 16:1295–305. doi: 10.1016/S1470-2045(15)00193-X 26384238

[52] Abaza Y Kantarjian H Garcia-Manero G Estey E Borthakur G Jabbour E. Long-term outcome of acute promyelocytic leukemia treated with all- trans-retinoic acid, arsenic trioxide, and gemtuzumab. Blood (2017) 129:1275–83. doi: 10.1182/blood-2016-09-736686 PMC5413297 28003274

[53] Kulkarni UP Selvarajan S Lionel S Prakash MA Palani HK Balasundaram N. Real world data with concurrent retinoic acid and arsenic trioxide for the treatment of acute promyelocytic leukemia. Blood Cancer J (2022) 12. doi: 10.1038/s41408-022-00619-3 PMC8803919 35102152

[54] Lancet JE Moseley AB Coutre SE DeAngelo DJ Othus M Tallman MS. A phase 2 study of ATRA, arsenic trioxide, and gemtuzumab ozogamicin in patients with high-risk APL (SWOG 0535). Blood Adv (2020) 4:1683–9. doi: 10.1182/bloodadvances.2019001278 PMC7189292 32330241

[55] Kingsley EC Durie BGM Garewal HS. Acute promyelocytic leukemia. Western J Med (1987) 146:322–7. PMC1307278 3472414

[56] Amadori S Testi AM Arico M Comelli A Giuliano M Madon E. Prospective comparative study of bone marrow transplantation and postremission chemotherapy for childhood acute myelogenous leukemia. J Clin Oncol (1993) 11:1046–54. doi: 10.1200/JCO.1993.11.6.1046 8501490

[57] Mann G Reinhardt D Ritter J Hermann J Schmitt K Gadner H. Treatment with all-trans retinoic acid in acute promyelocytic leukemia reduces early deaths in children. Ann Hematol (2001) 80:417–22. doi: 10.1007/s002770100304 11529468

[58] Hirota T Fujimoto T Katano N Tsurasawa M Eguchi H Nakadate N. [Treatment results of intermittent and cyclic regimen with ATRA and chemotherapy in childhood acute promyelocytic leukemia. children’s cancer and leukemia study group]. Rinsho Ketsueki (1997) 38:1177–82. 9423334

[59] Iarussi D Indolfi P Galderisi M Bossone E. Cardiac toxicity after anthracycline chemotherapy in childhood. Herz (2000) 25:676–88. doi: 10.1007/PL00001982 11141677

[60] Castaigne S Lefebvre P Chomienne C Suc E Rigal-Huguet F Gardin C. Effectiveness and pharmacokinetics of low-dose all-trans retinoic acid (25 mg/m2) in acute promyelocytic leukemia. Blood (1993) 82:3560–3. doi: 10.1182/blood.V82.12.3560.3560 8260694

[61] Testi AM Biondi A Lo Coco F Moleti ML Giona F Vignetti M. GIMEMA-AIEOP AIDA protocol for the treatment of newly diagnosed acute promyelocytic leukemia (APL) in children. Blood (2005) 106:447–53. doi: 10.1182/blood-2004-05-1971 15677559

[62] Ortega JJ Madero L Martín G Verdeguer A García P Parody R. Treatment with all-trans retinoic acid and anthracycline monochemotherapy for children with acute promyelocytic leukemia: a multicenter study by the PETHEMA group. J Clin Oncol (2005) 23:7632–40. doi: 10.1200/JCO.2005.01.3359 16234524

[63] Gregory J Kim H Alonzo T Gerbing R Woods W Weinstein H. Treatment of children with acute promyelocytic leukemia: results of the first north American intergroup trial INT0129. Pediatr Blood Cancer (2009) 53:1005–10. doi: 10.1002/pbc.22165 PMC3508725 19743516

[64] Bally C Fadlallah J Leverger G Bertrand Y Robert A Baruchel A. Outcome of acute promyelocytic leukemia (APL) in children and adolescents: an analysis in two consecutive trials of the European APL group. J Clin Oncol (2012) 30:1641–6. doi: 10.1200/JCO.2011.38.4560 22473162

[65] Creutzig U Zimmermann M Dworzak M Urban C Henze G Kremens B. Favourable outcome of patients with childhood acute promyelocytic leukaemia after treatment with reduced cumulative anthracycline doses. Br J Haematol (2010) 149:399–409. doi: 10.1111/j.1365-2141.2010.08107.x 20230404

[66] Sanz MA Montesinos P Vellenga E Rayón C De La Serna J Parody R. Risk-adapted treatment of acute promyelocytic leukemia with all-trans retinoic acid and anthracycline monochemotherapy: Long-term outcome of the LPA 99 multicenter study by the PETHEMA group. Blood (2008) 112:3130–4. doi: 10.1182/blood-2008-05-159632 18664623

[67] Testi AM Foa R Tomei G Coco Lo Biondi F A Pession A. GIMEMA-AIEOP AIDA protocols for the treatment of newly diagnosed acute promyelocytic leukemia (APL) in children: Analysis of 247 patients enrolled in two sequential Italian multicenter trials. Blood (2010) 116:871–1. doi: 10.1182/blood.V116.21.871.871

[68] Testi AM Pession A Diverio D Grimwade D Gibson B Cardoso de Azevedo A. Risk-adapted treatment of acute promyelocytic leukemia: Results from the international consortium for childhood APL. Blood (2018) 132:405–12. doi: 10.1182/blood-2018-03-836528 29789356

[69] Soignet SL Frankel SR Douer D Tallman MS Kantarjian H Calleja E. United states multicenter study of arsenic trioxide in relapsed acute promyelocytic leukemia. J Clin Oncol (2001) 19:3852–60. doi: 10.1200/JCO.2001.19.18.3852 11559723

[70] Fox E Razzouk BI Widemann BC Xiao S O’Brien M Goodspeed W. Phase 1 trial and pharmacokinetic study of arsenic trioxide in children and adolescents with refractory or relapsed acute leukemia, including acute promyelocytic leukemia or lymphoma. Blood (2007) 111:566–73. doi: 10.1182/blood-2007-08-107839 PMC2200837 17959855

[71] Cheng Y Zhang L Wu J Lu A Wang B Liu G. Long-term prognosis of childhood acute promyelocytic leukaemia with arsenic trioxide administration in induction and consolidation chemotherapy phases: A single-centre experience. Eur J Haematol (2013) 91:483–9. doi: 10.1111/ejh.12194 24033687

[72] Kutny MA Alonzo TA Gerbing RB Wang YC Raimondi SC Hirsch BA. Arsenic trioxide consolidation allows anthracycline dose reduction for pediatric patients with acute promyelocytic leukemia: Report from the children’s oncology group phase III historically controlled trial AAML0631. J Clin Oncol (2017) 35:3021–9. doi: 10.1200/JCO.2016.71.6183 PMC5590801 28767288

[73] Creutzig U Dworzak MN Bochennek K Faber J Flotho C Graf N. First experience of the AML-Berlin-Frankfurt-Münster group in pediatric patients with standard-risk acute promyelocytic leukemia treated with arsenic trioxide and all-trans retinoid acid. Pediatr Blood Cancer (2017) 64:1–4. doi: 10.1002/pbc.26461 28111878

[74] Li SY Lu Y Liu HC Gang EJ Le J Qian SY. Arsenic trioxide and all-trans retinoic acid in the treatment of children with newly diagnosed acute promyelocytic leukemia. Leuk Lymphoma (2021) 62:1267–70. doi: 10.1080/10428194.2020.1856832 33439058

[75] Hiroyuki T Shiro T Yuki Y Yuka IY Daisuke H Hiroshi M. Safety and efficacy of arsenic trioxide in the treatment of newly diagnosed pediatric acute promyelocytic leukemia: Results from the JPLSG AML-P13 study. ASH Meeting 2022 (2022).

[76] Rock N Mattiello V Judas C Huezo-Diaz P Bourquin JP Gumy-Pause F. Treatment of an acute promyelocytic leukemia relapse using arsenic trioxide and all-trans-retinoic in a 6-year-old child. Pediatr Hematol Oncol (2014) 31:143–8. doi: 10.3109/08880018.2013.876470 24498972

[77] Zheng H Jiang H Hu S Liao N Shen D Tian X. Arsenic combined with all-trans retinoic acid for pediatric acute promyelocytic leukemia: Report from the CCLG-APL2016 protocol study. J Clin Oncol (2021) 39:3161–70. doi: 10.1200/JCO.20.03096 PMC8478377 34077242

[78] Kutny MA Alonzo TA Abla O Rajpurkar M Gerbing RB Wang YC. Assessment of arsenic trioxide and all-trans retinoic acid for the treatment of pediatric acute promyelocytic leukemia: A report from the children’s oncology group AAML1331 trial. JAMA Oncol (2022) 8:79–87. doi: 10.1001/jamaoncol.2021.5206 34762093 PMC8587220

[79] Manara E Baron E Tregnago C Aveic S Bisio V Bresolin S. MLL-AF6 fusion oncogene sequesters AF6 into the nucleus to trigger RAS activation in myeloid leukemia. Blood (2014) 124(2):263–72. 10.1182/blood-2013-09-525741 24695851

